# Mothers had inadequate knowledge towards key essential nutrition action messages in mainly rural Northeast Ethiopia

**DOI:** 10.1017/jns.2021.10

**Published:** 2021-03-19

**Authors:** Bereket Gebremichael, Biruk Beletew Abate, Tewodros Tesfaye

**Affiliations:** 1 College of Health Science, Addis Ababa University, Addis Ababa, Ethiopia; 2 Department of Nursing, College of Health Science, Woldia University, Woldia, Ethiopia

**Keywords:** Essential nutrition actions, Knowledge, Attitude, Mothers, Rural Ethiopia, ANC, antenatal care, ANSD, Adequate Nutrition for Sustainable Development, EBF, exclusive breastfeeding, ENA, essential nutrition action, FAO, Food and Agriculture Organization, IRB, Institutional Review Board, PNC, postnatal care, SPSS, Statistical Package for Social Science, WHO, World Health Organization

## Abstract

Essential nutrition action (ENA) is one of the most effective preventive actions for combating nutritional problems in young children. There is, however, a paucity of evidence about mother's knowledge and attitude regarding key ENA messages. The objective of the present study was to assess the knowledge and attitude of mothers towards key ENA messages and associated factors. A representative sample of 563 mothers of children from birth up to 24 months in mainly rural North Ethiopia was included in the study. The findings showed that 66⋅4 % of the mothers have a good knowledge and 68⋅9 % have a good attitude. In the multivariable analysis using logistic regression, mothers who attended secondary school or higher education were six times more likely to have a good knowledge (AOR 6⋅1; CI 2⋅945, 12⋅719) compared with those who are illiterate. Besides, women who resided in an urban area (AOR 2⋅2; CI 1⋅14, 4⋅25), attended antenatal care (ANC) visits (AOR 3⋅7; CI 2⋅421, 5⋅742), attended postnatal care (PNC) visits (AOR 2⋅2; CI 1⋅37, 3⋅4) and heard nutritional-related information (AOR 1⋅9; CI 1⋅14, 3⋅49) were found to have a good knowledge. On the other hand, mothers who attended ANC visits were almost four times (AOR 3⋅9; CI 2⋅7, 5⋅8) more likely to have a good attitude towards key ENA. Mothers who delivered at health institutions and who attended PNC visits were also more likely to have a good attitude. In conclusion, the present study determined the level of knowledge and attitudes of mothers about ENA and several factors that influence mother's knowledge and attitude regarding ENA.

## Background

The first 1000 days of life (from conception to a child's second birthday) plays a crucial role in the child's health and survival^(1)^. It is during this critical period that a child's brain and body develop^(2,3)^. Proper nutrition during this period plays an integral role in enabling a child to grow and develop. However, millions of children are affected by poor nutrition during this critical period^(4)^.

Worldwide, maternal and child undernutrition is responsible for 10 % of disease burden and over 30 % of childhood death^(5)^. More than 17 million children do not reach their full potential due to inadequate nutrition in the early months of their lives^(3,6)^. Over seven million children die every year before celebrating their fifth birthday and malnutrition accounts for more than 35 % of the total death. Developing countries including Ethiopia contribute more than 95 % of these deaths^(7)^. The devastating impact of undernutrition spans generations as chronically malnourished children are more likely to become shorter adults and give birth to low birth weight offspring^(6)^. Children aged less than 2 years of age are especially vulnerable since the consequence of malnutrition at this particular age are mostly irreversible^(6)^.

Different strategies have been proven to effectively improve the nutritional status of children^(8)^. Essential nutrition action (ENA) is one of the approaches to expand the coverage of seven affordable and evidence-based nutrition actions to improve the nutritional status of women and children, particularly those less than 2 years of age. It incorporates seven messages such as exclusive breastfeeding (EBF), complementary feeding, nutritional care of sick children, nutrition for women during pregnancy and lactation, prevention of vitamin A deficiency, prevention of anaemia and prevention of iodine deficiency^(2,3,9)^. ENA focus on integrating different nutritional interventions within commonly available health facilities. The main goal of this approach is to enhance the quality of nutritional services and to achieve positive changes in family-based feeding and caring behaviours^(9)^.

Studies have shown that the prevalence of inadequate infant and young child feeding (IYCF) and micro-nutrients deficiency (MND) is high in Ethiopia^(10,11)^. The high prevalence could be mainly due to a lack of awareness about proper feeding and other malnutrition preventive measures that are pillars of ENA. The direct caregiver of the child should have the right kind of knowledge and attitude^(12)^. Moreover, if implemented properly, ENA could reduce nutrition-related disease and death burden by 25 %^(3)^. Furthermore, in the rural area, where there is a lack of resources, child nutrition will be more hampered by a lack of knowledge. However, there is a paucity of evidence that investigated knowledge and attitude about ENA messages. Therefore, the purpose of the present study was to assess the knowledge and attitudes of mothers regarding key ENA messages and factors associated with them in mainly rural northern Ethiopia.

## Methods

### Study setting, design and Population

This is a community-based cross-sectional study conducted in predominantly rural Woreda in the Northeast part of Ethiopia, known as Wereilu. The woreda is 492 km north of Addis Ababa, the capital city of Ethiopia. It is comprised of 24 kebeles (20 rural and 4 urban). The woreda has more than 15 health facilities among which 5 health centres and 1 district hospital are run by the government^(13)^.

The present study is based on data collected for Adequate Nutrition for Sustainable Development (ANSD) project. The project aims to assess the awareness of mothers of young children less than 24 months of age about IYCF to develop tailored interventions. Part of the project finding is published previously and the detail of methodology is discussed in the publication^(14)^.

A representative sample of 563 study participants (mothers of children birth up to 24 months of age) were interviewed in April 2018.

### Sample size and sampling procedure

A single population proportion formula was used to determine the sample size. We used the proportion of complementary feeding practice in a rural community of Amhara Regional State, Ethiopia (*P* = 56⋅5 %) from a previous study, which is one of the components of ENA messages^(15)^. A 95 % confidence level and 5 % margin of error was used. Furthermore, we added a design effect of 1⋅5 and 5 % non-response rate which gave a total sample size of 563.

From the total 24 kebeles in the woreda, 9 kebeles (7 rural and 2 urban kebeles) were randomly selected using a simple random sampling technique. To proportionally allocate the estimated sample size to each kebele, using probability proportional to size method. Samples were proportionally allocated to each kebele based on the population size of children under 2 years in the respective kebeles. To select the study participants, a systematic random sampling technique was employed. In households that had more than one eligible child, one child was randomly selected using a lottery method. If nobody in the household was available at the time of the interview, households were revisited three times. After three attempts, those who were unavailable were considered as non-respondent.

### Data collection tool and procedure

A structured, pretested and interviewer-administered questionnaire was used to collect the data. The questionnaire was adapted from the World Health Organization (WHO) and the Food and Agriculture Organization (FAO) guidelines and questionnaires^(3,16)^. The questionnaire was first developed in the English language and translated into the local language (Amharic) by language and nutrition experts. To check for consistency, it was translated back to the English language by an independent translator. Seven diploma nurses were recruited to collect data and two BSc nurses supervised the data collection process.

The questionnaire contains a question on nine components, such as socio-demographic variables, EBF, complementary feeding, feeding of sick child, nutrition during pregnancy and lactation, prevention of Vitamin A deficiency, prevention of anaemia, prevention of iodine deficiency disorder and access to nutritional information which are the components of ENA. The interview was conducted with the mothers at their homes. The interview took approximately 45 min to 1 h to complete.

### Data quality control

A 2-d training was conducted for data collectors and supervisors on the data collection techniques and ethical aspects related to the interview. The data collection tool was pretested in 5 % of the total sample size (*n* 28) and necessary adjustments were made to the tool. The pretest data were not included in the final analysis.

Daily close supervision of data collectors was conducted to ensure the quality of the data. Supervisors reviewed the collected data in the field for checking the completeness and consistency of the answers and necessary corrections were made in the field. Data entry was done daily and missing data were identified. Incorrectly filled information or questionnaires that missed major components were excluded from the analysis.

### Data processing and analysis

Data entry and analysis were conducted using statistical software EPI-data Version 4.2 and Statistical Package for Social Science (SPSS) version 24. The analysis included descriptive statistics to characterise the study population. Mean scores were calculated to report the knowledge and attitude level of study participants. Respondents who scored mean and above the mean for knowledge and attitude questions were categorised as having good knowledge or good attitude, respectively. Those who scored below the mean score were categorised as having poor knowledge or poor attitude.

Explanatory variables, which showed associations with the outcome variable at *P* < 0⋅25 in the bivariate analysis, were included in the final model building for the logistic regression model to adjust for possible confounders. Odds ratio (OR) with 95 % confidence interval (CI) and *P*-value was computed to assess the existence and strength of the association between dependent and independent variables. In the final model, variables with a *P* < 0⋅05 were considered statistically significant.

### Ethics approval and consent to participate

This study was conducted according to the guidelines laid down in the Declaration of Helsinki and all procedures involving human subjects/patients were approved by the Addis Ababa University, Institutional Review Board [Protocol number-AAU/CHS/CHNSG02/2018]. Written informed consent was obtained from all subjects/patients.

## Result

### Characteristics of the study subjects

Among the selected 563 study participants, 550 completed the study resulting in a response rate of 97⋅7 %. About 34 and 38⋅7 % of the mothers were aged between 25–29 and 30–34 years, respectively. Regarding the educational status of the mothers, more than 40 % were either illiterate or did not have any formal education. In terms of residence and wealth index, 26⋅5 and 60⋅7 % were urban residents and in the middle wealth tertile. Most of the children (69 %) were younger than 12 months, and 58⋅9 % were male by gender (Table 1).

**Table 1.** Socio-demographic characteristics of study participants, Northeast Ethiopia, 2018

Socio-demographic and related variables	Frequency (*N* 550)	Percent
Age of the mother		
<20 years	11	2⋅0
20–24 years	99	18⋅0
25–29 years	187	34⋅0
30–34 years	213	38⋅7
>35 years	40	7⋅3
Religion		
Orthodox	224	40⋅7
Protestant	148	26⋅9
Muslim	173	31⋅5
Catholic	5	0⋅9
Marital status		
Single	39	7⋅1
Married	482	87⋅6
Separated	28	5⋅1
Divorced	1	0⋅2
Educational status of the mother		
Cannot read and write	107	19⋅5
Can read and write	180	32⋅7
Primary	162	29⋅5
Secondary	80	14⋅5
Higher education	21	3⋅8
Occupation of the mother		
Government employed	52	9⋅5
Employed in private sector	1	0⋅2
Merchant	17	3⋅1
Residence		
Urban	146	26⋅5
Rural	404	73⋅5
Wealth index		
Lowest tertile	164	29⋅8
Meddle tertile	334	60⋅7
Highest tertile	52	9⋅5
Number of family members		
<4	106	19⋅3
4–5	233	42⋅4
6–8	122	22⋅2
≥9	89	16⋅2
Parity		
Multipara	427	77⋅6
Primipara	123	22⋅4
Background of the child		
Age of the child		
<6 months	181	32⋅9
6–12 month	206	37⋅5
12–18 month	106	19⋅3
18–24 month	57	10⋅3
Sex		
Male	324	58⋅9
Female	226	41⋅1

### Knowledge and attitudes related to key ENA messages

From the total study participants who responded to questions to assess knowledge and attitude towards key ENA messages, 365 (66⋅4 %) and 379 (68⋅9 %) respondents had a good knowledge and a good attitude, respectively.

### Information access related to nutrition

Access to nutrition-related information was assessed. Most of the respondents 460 (75⋅6 %) had heard messages about maternal and children feeding practices. The main source of information was nurses, doctors, health extension workers or media. About 44⋅7 % of respondents had received the information during pregnancy and 21⋅4 % during the postnatal period. Delivery time, immunisation, and routine child visits were also mentioned as timing when the information was received by participants.

### Factors associated with knowledge towards key ENA messages

Based on the output of multivariable logistic regression, many explanatory variables like maternal educational status, attendance of ANC follow-up, urban residence, and access to nutrition-related information were found to determine knowledge about ENA. Mothers who attended primary school were almost three times [AOR 2⋅8; 95 % CI (1⋅650, 4⋅801)] and those who attended secondary school and above were six times [AOR 6⋅1; 95 % CI (2⋅945, 12⋅719)] more likely to have a good knowledge regarding key ENA messages as compared with illiterate mothers. Urban residence was also associated with good knowledge of key ENA messages. Respondents who resided in urban areas were 2⋅2 times [AOR 2⋅2; 95 % CI (1⋅14, 4⋅25)] more likely to have a good knowledge than their counterparts from rural areas. The odds of good knowledge were higher among mothers who had ANC follow-up. Those who attended an ANC follow-up were almost four times [AOR 3⋅7; 95 % CI (2⋅421, 5⋅742)] more likely to have a good knowledge when compared with mothers who did not attend. In addition, mothers who had received nutritional-related information were two times [AOR 1⋅9; 95 % CI (1⋅14, 3⋅49)] more likely to have a good knowledge than those who did not receive that information (Table 2).

**Table 2.** Factors associated with knowledge of key ENA messages among study participants, Northeast Ethiopia, 2018

Variables	Level of knowledge (*N* 550)	
	Good Poor		Odd Ratios	
	Frequency (%)	Frequency (%)	COR (95 % CI)	AOR (95 % CI)	*P*-value
Educational status of the mother					
Illiterate	146(40⋅0)	141(76⋅2)	1	1	
Primary	131(35⋅9)	31(16⋅8)	4⋅0(2⋅59–6⋅4)	2⋅8(1⋅65–4⋅8) *	<0⋅001
Secondary and above	88(24⋅1)	13(7⋅0)	6⋅5(3⋅5–12⋅2)	6⋅1(2⋅9–12⋅7)*	<0⋅001
Residence					
Urban	100(27⋅4)	15(8⋅1)	4⋅2(2⋅4–7⋅6)	2⋅2(1⋅14–4⋅25)*	0⋅019
Rural	265(72⋅6))	170(91⋅9	1	1	
ANC follow-up					
Yes	250(68⋅5)	53(28⋅6)	5⋅4(3⋅67–7⋅98)	3⋅7(2⋅42–5⋅74)*	<0⋅001
No	115(31⋅5)	132(71⋅4)	1	1	
PNC follow-up					
Yes	195(53⋅4)	52(28⋅1)	1	1	
No	170(46⋅6)	133(71⋅9)	2⋅9(2⋅0–4⋅29)	2⋅2(1⋅37–3⋅4)*	0⋅001
Hear nutritional-related information					
Yes	324(88⋅8)	123(66⋅5)	3⋅9(2⋅5–6⋅2)	1⋅9(1⋅14–3⋅49)*	015
No	41(11⋅2)	62(33⋅5)	1	1	.

CI, confidence interval; COR, crude odds ratio; AOR, adjusted odds ratio; ORs, odds ratios; ANC, antenatal care; PNC, postnatal care.

* *P*-value < 0⋅05.

### Factor associated with attitudes towards key ENA messages

Several explanatory variables like sex of the child, place of delivery, attending antenatal care (ANC) follow-up or postnatal care (PNC) follow-up were found to determine attitudes towards key ENA messages. Mothers who had a male child were two times [AOR 2⋅1; 95 % CI (1⋅36, 3⋅18)] more likely to have a good attitude towards key ENA messages than mothers of girls. Mothers who delivered at health facilities were almost two times [AOR 1⋅7; 95 % CI (1⋅045, 2⋅995)] more likely to have a favourable attitude towards key ENA messages than those who delivered at home. Also, mothers who attended ANC or PNC follow-up were 2⋅4 and 1⋅7 times more likely to have a good attitude towards key ENA messages than their counterparts, respectively. Moreover, mothers with good knowledge regarding key ENA messages were 2⋅5 times [AOR 2⋅5; 95 % CI (1⋅6, 4⋅08)] more likely to have a favourable attitude than those with poor knowledge (Table 3).

**Table 3.** Factors associated with attitude towards key ENA messages among study participants, Northeast Ethiopia, 2018

Variables	Level of attitude (*N* 550)		Odd Ratios		*P*-value
	Good Poor				
	Frequency (%)	Frequency (%)	COR (95 % CI)	AOR (95 % CI)	
Educational status of the mother					
Illiterate	162(42⋅7 %)	125(73⋅1)			
Primary	133(35⋅1)	29(17⋅0)	3⋅5(2⋅2–5⋅6)	1⋅7(0⋅99–3⋅13)	0⋅054
Secondary and above	84(22⋅2)	17(9⋅9)	3⋅8(2⋅15–6⋅75)	2⋅0(0⋅98–4⋅2)	0⋅057
Residence					
Urban	99(26⋅1)	16(9⋅4)	3⋅4(1⋅9–6⋅0)	1⋅5(0⋅81–2⋅94)	0⋅189
Rural	280(73⋅9)	155(90⋅6)	1	1	
Monthly income					
Higher	128(33⋅8)	36(21⋅1)	2⋅0(1⋅0–4⋅0)	1⋅5(0⋅71–3⋅50)	0⋅263
Medium	218(57⋅5)	116(67⋅8)	1⋅0(0⋅5–1⋅9)	1⋅2(0⋅62–2⋅61)	0⋅509
Low	33(8⋅7)	19(11⋅1)	1	1	
Sex of the child					
Male	245(64⋅6)	79(46⋅2)	2⋅1(1⋅47–3⋅07)	2⋅0(1⋅36–3⋅18)*	0⋅001
Female	134(35⋅4)	92(53⋅8)	1	1	
Place of birth					
Health institution	258(68⋅1)	132(77⋅2)	1⋅6(1⋅05–2⋅41)	1⋅7(1⋅04–2⋅99)*	0⋅034
Home	121(31⋅9)	39(22⋅8)	1	1	
ANC follow-up					
Yes	248(65⋅4)	55(32⋅2)	3⋅9(2⋅7–5⋅8)	2⋅4(1⋅53–3⋅86)*	0⋅000
No	179(47⋅2)	124(72⋅5)	1	1	
Hear nutritional-related information					
Yes	318(83⋅9)	129(75⋅4)	1⋅7(1⋅09–2⋅6)	1⋅4(0⋅82–2⋅49)	0⋅210
No	61(16⋅1)	42(24⋅6)	1	1	
Knowledge level					
Good knowledge	294(77⋅6) 5)	71(41⋅5)	4⋅8(3⋅3–7⋅18)	2⋅5(1⋅6–4⋅08)*	0⋅000
Poor knowledge	85(22⋅4)	100(58⋅5)	1	1	

CI, confidence interval; COR, crude odds ratio; AOR, adjusted odds ratio; ORs, odds ratios; ANC, antenatal care.

* *P*-value < 0⋅05.

## Discussion

This community-based cross-sectional study assessed the knowledge and attitudes of mothers of children from delivery to 24 months of age. The present study also aimed to identify factors associated with the knowledge and attitudes towards key ENA messages. Based on the present study, the overall prevalence of good knowledge and good attitude was not sufficient. Various factors like maternal educational status, place of residence, ANC or PNC follow-up, and receiving nutrition-related information were factors identified to affect knowledge. Besides, the child's sex, place of delivery, ANC or PNC follow-up, and knowledge about ENA were factors associated with attitudes towards key ENA messages.

The ENA framework is an approach for managing the advocacy, planning and delivery of an integrated package of interventions to reach a wide coverage to achieve a public health impact. This can be realised through training of health personal, integrating the service in the healthcare system and improving knowledge, attitudes, and practices of mothers in order to seek the services^(17)^. In the present study, 66⋅4 % of the study participants had a good knowledge of key ENA messages. This is below the desired universal coverage of the service. Our finding is also lower than what other studies found related to knowledge of mothers towards different ENA messages^(18–20)^. The possible justification for this variation could be differences in study settings, economic and demographic characteristics, and access to nutrition education because our study was conducted in mostly rural areas.

Regarding attitude, 68⋅9 % of the mothers had a good attitude towards key ENA messages. This finding was higher than a study conducted in Ambo, Ethiopia, where a good attitude towards key ENA messages was only 32 %^(21)^. The possible explanation for this difference could be the time difference between the two studies. On the other hand, our finding was lower than other studies in different parts of Ethiopia that assessed attitude towards EBF which is one of the ENA messages^(19,22)^. This might be because our study computed a sum score regarding attitudes of the mothers about the seven components of key ENA messages but not based on a single ENA component.

Regarding factors associated with knowledge about key ENA messages, the odds of good knowledge were higher among mothers who had primary and secondary or higher education compared with illiterate mothers. The finding was comparable with previous studies conducted in Northern Ethiopia^(15,23,24)^. The possible reason behind higher knowledge among educated mothers could be due to literate mothers can easily access nutrition-related information from different media channels (both printed and audiovisual).

Mothers who delivered in institutions had better access to information related to different child care practices including components of ENA. The present study revealed that mothers who delivered at health institutional had higher odds of positive attitude towards key ENA messages when compared with mothers who delivered at home. This was also shown by research conducted in another part of Ethiopia^(25)^.

According to the present study, there was a significant association between knowledge regarding key ENA messages and PNC service utilisation. Similar findings were also reported by previous studies conducted in different parts of Ethiopia^(15,24,26)^. The possible justification for good knowledge among those who received PNC service could be health and nutrition-related information and counselling are given as part of these services. In addition, mothers who received nutrition information were found to have a good knowledge of key ENA messages.

Concerning the sex of the child, better attitudes towards key ENA messages were observed among mothers of male children compared with mothers of girls. One possible explanation for this could be that traditionally in the study area, it is believed that male children should be fed more than female children to become stronger.

Place of residence was also one of the factors associated with knowledge about key ENA messages. Mothers who resided in urban areas had better knowledge about key ENA messages compared with those who lived in rural areas. The finding was comparable with another study finding in Northwest Ethiopia^(27)^. This could be explained by the fact that urban mothers have better access to education, have a higher socioeconomic status and more access to nutrition information than rural mothers. On the other hand, rural women might be influenced by cultural beliefs and misconceptions as a result of limited access to health information.

Pregnancy is considered to be an important window of opportunity in providing health and nutrition-related information. Women who attended ANC would get more information about various health issues that could influence their attitude towards health including ENA. Similarly, in our study, those mothers who had ANC follow-up were found to have a good attitude towards key ENA messages than mothers who did not attend ANC follow-up. This finding was similar to a study in Northeast Ethiopia^(15)^.

## Limitations

Despite several strengths, the present study has certain limitations. First, making causal inferences between dependent and independent variables is not possible due to the nature of the study design. Second, the response might be affected by social desirability and recall bias. Lastly, only interviewing mothers might have limited the information since other caregivers, such as fathers or grandmothers might have added different perspectives.

## Conclusion and recommendation

The present study tried to determine the level of knowledge and attitudes related to key ENA messages among mothers’ children under 2 years of age in mainly rural Northeast Ethiopia. The knowledge and attitudes of mothers towards key ENA messages were not sufficient. Several variables were identified to determine both knowledge and attitudes. Maternal educational status, place of residence, ANC or PNC follow-up, and receiving nutrition information were identified associated factors with knowledge about ENA messages. Moreover, child's sex, delivery at health facility, ANC or PNC follow-up, and knowledge about ENA were identified associated factors with attitudes towards ENA messages. Since mothers who did not use maternal and child health (MCH) services such as ANC, delivery and, PNC were more likely to have a poor knowledge and unfavourable attitudes towards ENA, health education efforts should be expanded about the use of MCH services concentrating on uneducated and rural women. This creates a good opportunity for providing awareness creation about various health and nutrition issues. Moreover, community-based nutrition education should be enhanced because mothers who have information regarding ENA had a favourable attitude towards it.
